# Comparing deep learning and handcrafted radiomics to predict chemoradiotherapy response for locally advanced cervical cancer using pretreatment MRI

**DOI:** 10.1038/s41598-024-51742-z

**Published:** 2024-01-12

**Authors:** Sungmoon Jeong, Hosang Yu, Shin-Hyung Park, Dongwon Woo, Seoung-Jun Lee, Gun Oh Chong, Hyung Soo Han, Jae-Chul Kim

**Affiliations:** 1 Department of Medical Informatics, School of Medicine, Kyungpook National University, Daegu, Republic of Korea; 2 Research Center for Artificial Intelligence in Medicine, Kyungpook National University Hospital, Daegu, Republic of Korea; 3 Department of Radiation Oncology, School of Medicine, Kyungpook National University, Daegu, Republic of Korea; 4 Department of Radiation Oncology, Kyungpook National University Hospital, Daegu, Republic of Korea; 5 Cardiovascular Research Institute, School of Medicine, Kyungpook National University, Daegu, Republic of Korea; 6 Department of Gynecology, School of Medicine, Kyungpook National University, Daegu, Republic of Korea; 7 Clinical Omics Research Center, School of Medicine, Kyungpook National University, Daegu, Republic of Korea; 8 Department of Physiology, School of Medicine, Kyungpook National University, Daegu, Republic of Korea

**Keywords:** Oncology, Cancer imaging, Gynaecological cancer, Tumour heterogeneity

## Abstract

Concurrent chemoradiotherapy (CRT) is the standard treatment for locally advanced cervical cancer (LACC), but its responsiveness varies among patients. A reliable tool for predicting CRT responses is necessary for personalized cancer treatment. In this study, we constructed prediction models using handcrafted radiomics (HCR) and deep learning radiomics (DLR) based on pretreatment MRI data to predict CRT response in LACC. Furthermore, we investigated the potential improvement in prediction performance by incorporating clinical factors. A total of 252 LACC patients undergoing curative chemoradiotherapy are included. The patients are randomly divided into two independent groups for the training (167 patients) and test datasets (85 patients). Contrast-enhanced T1- and T2-weighted MR scans are obtained. For HCR analysis, 1890 imaging features are extracted and a support vector machine classifier with a five-fold cross-validation is trained on training dataset to predict CRT response and subsequently validated on test dataset. For DLR analysis, a 3-dimensional convolutional neural network was trained on training dataset and validated on test dataset. In conclusion, both HCR and DLR models could predict CRT responses in patients with LACC. The integration of clinical factors into radiomics prediction models tended to improve performance in HCR analysis. Our findings may contribute to the development of personalized treatment strategies for LACC patients.

## Introduction

Cervical cancer is the fourth most common cancer and the fourth leading cause of cancer-related deaths in women worldwide, as reported in the 2020 Global Cancer Statistics Report^1^. Concurrent chemoradiotherapy (CRT) is the standard treatment for locally advanced diseases, while patients with early lesions can be treated with surgery. However, the current CRT regimen, consisting of external beam radiotherapy (EBRT) and intracavitary brachytherapy (ICR) with concurrent cisplatin, is quite uniform despite the substantial diversity of treatment responsiveness^2,3^. A reliable tool for predicting CRT responses may help identify patients who are most likely to have a good response and enable personalized treatment according to each patient’s given probability of treatment success.

Recently, considerable advancements have been achieved in medical imaging, which has resulted in the emergence of computational techniques that extract information hidden from the human eye. Radiomics, the extraction of quantitative features from medical images, has emerged as a promising tool for assisting clinical care, particularly in cancer diagnosis and prognosis prediction. Conventional handcrafted radiomics (HCR) and deep learning-based radiomics (DLR) are currently available for radiomic analysis. In contrast to HCR, which requires ROI segmentation, feature extraction, and feature selection, DLR can omit some of these steps in its pipeline; thus, it requires relatively less time and effort for both feature extraction and selection processes, and simplifying the pipeline.

Our study aimed to predict CRT response in locally advanced cervical cancer (LACC) with both HCR and DLR analysis using pretreatment MR scans. By comparing the prediction performance of these models, we aimed to determine which model performed better. Additionally, we investigated the potential improvement in prediction performance by incorporating clinical data into radiomics models.

## Results

### Patient characteristics

The median age at diagnosis for all included patients was 57 years (range: 23–86 years). The FIGO stages were IIB, IIIA, IIIB, IIIC1, IIIC2, and IVA in 87 (34.5%), 1 (0.4%), 9 (3.6%), 121 (48.0%), 33 (13.1%), and 1 (0.4%) patient (s), respectively^4^. Overall, 77.4% of patients achieved complete remission at 3 months after CRT. The patient characteristics in the training and test datasets are presented in Table 1. The characteristics of patients in the training and test datasets were not significantly different in terms of age, tumor size, FIGO stage, human papilloma virus (HPV) infection status, and CRT response.

**Table 1:** Patient characteristics of the 252 patients.

Characteristic	Training set	Test set	*p*-value
Age			0.755
Median (range)	57 (24–86)	57 (23–84)	
Pathology			0.779
Squamous cell carcinoma (SCC)	152 (91.0%)	79 (92.9%)	
Non-SCC	15 (9.0%)	6 (7.1%)	
Tumor size (mm)			0.323
< 50	93 (55.7%)	44 (51.8%)	
≥ 50	74 (44.3%)	41 (48.2%)	
FIGO Stage			0.676
IIB-IIIB	133 (79.6%)	65 (76.5%)	
IIIC1-IVA	34 (20.4%)	20 (23.5%)	
HPV infection status			0.267
Positive	94 (56.3%)	39 (45.9%)	
Negative	25 (15.0%)	14 (16.5%)	
Unknown	48 (28.7%)	32 (37.6%)	
Chemoradiotherapy response			0.931
Complete remission	130 (77.8%)	65 (76.5%)	
Non-complete remission	37 (22.2%)	20 (23.5%)	

*HPV* human papilloma virus.

### Handcrafted radiomics model performance

**Figure 1:**
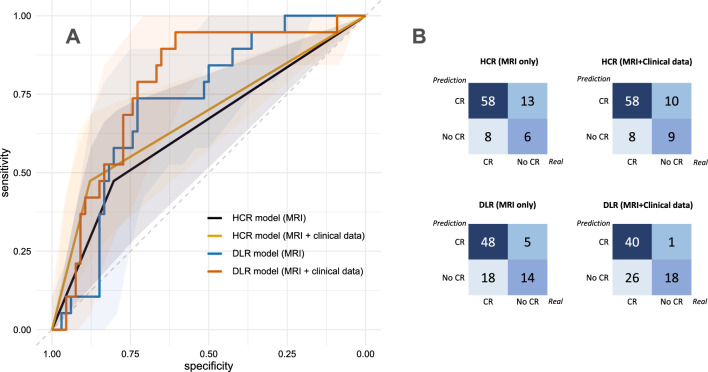
(**A**) Receiver operating characteristic (ROC) curves and (**B**) confusion matrices of the prediction models constructed using handcrafted radiomics (HCR) and deep-learning radiomics (DLR) for predicting complete response (CR) after chemoradiotherapy in the test dataset.

By applying logistic regression and recursive feature elimination, 20 imaging features were selected for the analysis (Supplementary Table 1). The support vector machine (SVM) classifier used these 20 imaging features for the binary classification of CRT response (complete response or not). The AUC was 0.597 (95% CI 0.513–0.763) and balanced accuracy of the classification was 0.598 in the test dataset (Fig. 1 and Table 2). When adding three clinical factors (tumor size, FIGO stage, and HPV status) into the SVM modeling, the SVM classifier exhibited the AUC of classification of 0.676 (95% CI 0.554–0.798) and balanced accuracy of 0.676 in the test dataset. The model incorporating clinical factors showed marginally significant improvement compared to the model using only MRI data (*p*-values; 0.096 for the DeLong test, 0.085 for the net reclassification improvement (NRI), and 0.092 for the integrated discrimination improvement (IDI)).

**Table 2:** Performance of HCR and DLR classifiers for predicting chemoradiotherapy response in test dataset.

Classifier	AUC	Balanced accuracy		Sensitivity		Specificity	PPV		NPV	MCC
HCR (MR)	0.597	0.598		0.316		0.879	0.429		0.817	0.263
HCR (MR + CF)	0.676	0.676		0.474		0.879	0.529		0.853	0.367
DLR (MR)	0.721	0.732		0.737		0.727	0.438		0.906	0.399
DLR (MR + CF)	0.782	0.777		0.947		0.606	0.409		0.976	0.461
Comparisons between prediction models										
			Delong test (*p*-value)		NRI [95% CI] (*p*-value)			IDI [95% CI] (*p*-value)		
HCR (MR) versus DLR (MR)			0.096		0.270 [− 0.022–0.561] (0.070)			0.270 [− 0.029–0.568] (0.077)		
HCR (MR + CF) versus DLR (MR + CF)			0.176		0.201 [− 0.062–0.464] (0.134)			0.201 [− 0.068–0.470] (0.142)		
HCR (MR) versus HCR (MR + CF)			0.092		0.158 [− 0.022–0.337] (0.085)			0.158 [− 0.026–0.342] (0.092)		
DLR (MR) versus DLR (MR + CF)			0.223		0.089 [− 0.127–0.306] (0.419)			0.089 [− 0.132–0.311] (0.429)		

*AUC* area under curve, *PPV* positive predictive value, *NPV* negative predictive value, *MCC* Matthew’s correlation coefficient, *HCR* handcrafted radiomics, *MR* magnetic resonance image, *CF* clinical factors, *DLR* deep learning radiomics, *NRI* net reclassification improvement, *IDI* integrated discrimination improvement.

### Deep learning model performance

The DLR model using MRI data (DLR (MR)) performed better than the HCR model (Fig. 1 and Table 2), with AUC of 0.721 (95% CI 0.617–0.847) and a balanced accuracy of 0.732. When clinical factors were incorporated into DLR models (DLR (MR + CF)), predictive performance was further improved, with AUC of 0.782 (95% CI 0.658–0.843) and a balanced accuracy of 0.777. However, there was no statistically significant difference between DLR (MR) and DLR (MR + CF) models.

**Figure 2:**
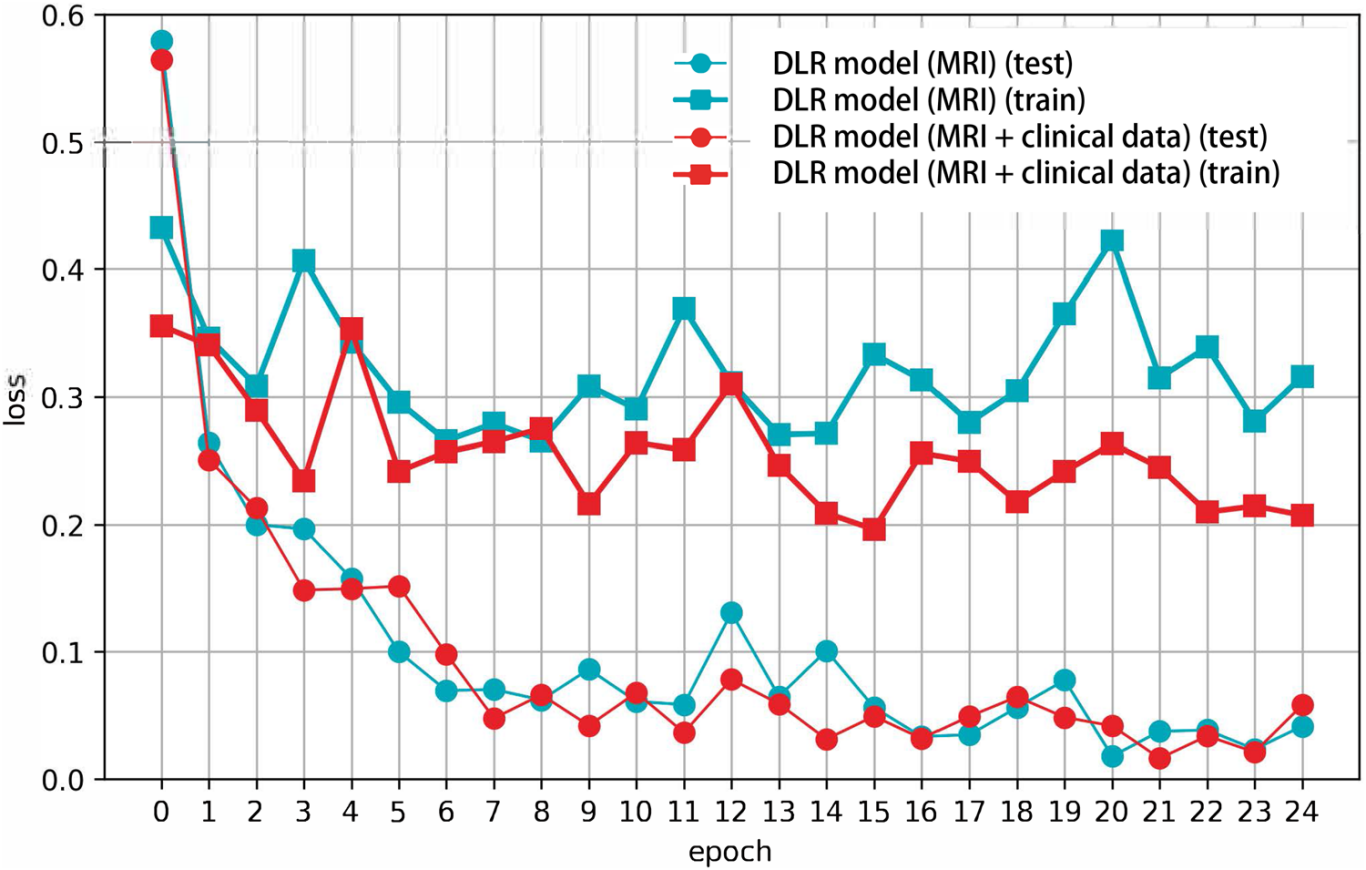
Training (square) and testing (circle) losses for the DLR model using MRI data (cyan lines) and the model using both MRI and clinical factor data (red lines). Both training and testing losses are smaller in the model using MRI and clinical factor data, as compared to those using only MRI data.

**Figure 3:**
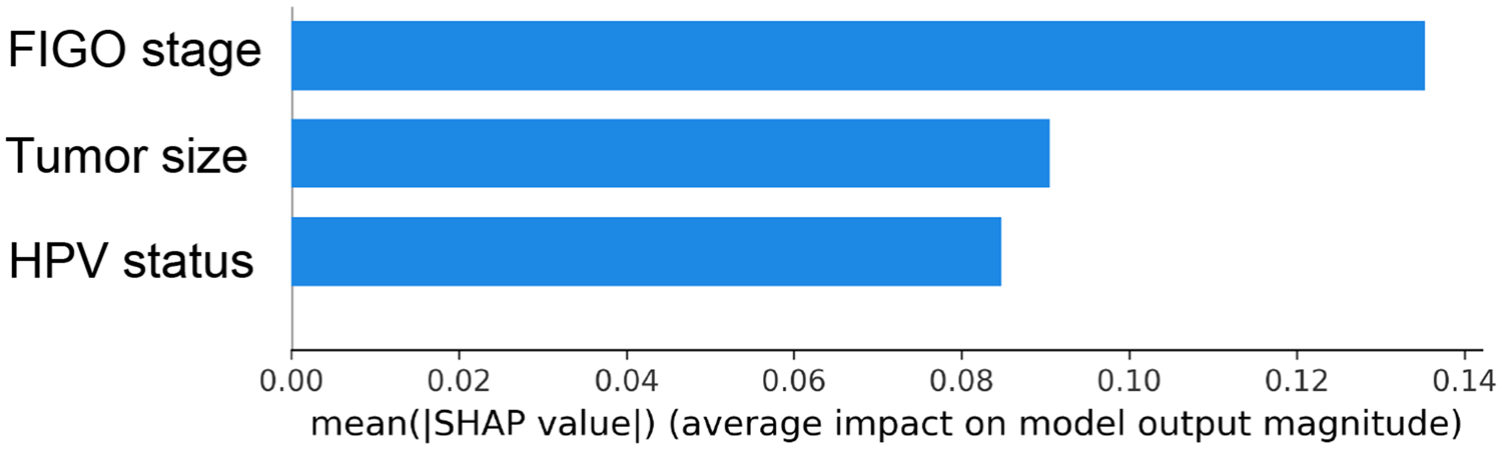
Each bar represents the absolute value of the SHapley Additive exPlanations (SHAP) analysis, which represents the average marginal contribution of each clinical factor to the total prediction.

Figure 2 depicts the training and testing loss for DLR models with or without clinical factors. DLR models with clinical factors exhibited smaller loss values for both training and test datasets compared to those in the models without clinical factors. Through the SHapley Additive exPlanations (SHAP) analysis, which employs game theory to measure the contribution of each feature in the DLR model, it was found that the FIGO stage contributed the most substantially among clinical factors, followed by tumor size and HPV infection status (Fig. 3).

### Comparison between radiomics and deep learning model

Comparing HCR and DLR models using MRI data, ROC analysis revealed that the DLR model tended to improve performance in terms of predicting response after CRT (0.597 vs. 0.721, *p* = 0.096). In addition, the NRI and IDI analyses revealed a marginal improvement in the accuracy of the association with response after CRT in the DLR model (NRI = 0.270, *p* = 0.070; IDI = 0.270, *p* = 0.077). However, when comparing HCR and DLR models using MRI data and clinical factors, neither ROC analysis nor NRI/IDI exhibited a significant difference between the two models. The sensitivity, which refers to the capability of a model to correctly predict cases not achieving a complete response after CRT, was 0.316 without clinical data and 0.474 with clinical data for the HCR models. For the DCR models, sensitivity was 0.737 without clinical data and 0.947 with clinical data.

### Potential factors related to CRT response

None of the potential factors (tumor size, HPV status, FIGO stage, age, pathology, lymph node metastasis status, and parametrial invasion) were related to CRT response (Supplementary Table 2).

### Uncertainty quantification

The experimental results comparing the Brier scores for HCR and DLR in test dataset are shown in Table 3. Our result shows that DLR had a lower Brier score than HCR both when using only MRI data (0.214 vs. 0.246) and when using both MRI data and clinical factors (0.193 vs. 0.250).

**Table 3:** Brier scores of HCR and DLR classifiers for uncertainty quantification.

Classifier	Brier score
HCR (MR)	0.246
HCR (MR + CF)	0.250
DLR (MR)	0.214
DLR (MR + CF)	0.193

*HCR* handcrafted radiomics, *MR* magnetic resonance image, *CF* clinical factors, *DLR* deep learning radiomics.

## Discussion

Predicting CRT response before treatment is of clinical significance. Patients predicted to have a poor response can benefit from dose escalation or alternative treatments. Conversely, those expected to have a good response might be candidates for de-intensified treatment, thereby reducing the risk of treatment-related side effects. In other words, personalized medicine can be provided to patients with LACC. We compared prediction performance of HCR and DLR models. Our study demonstrated that DLR models generally outperformed the HCR models in predicting CRT response in LACC patients, although the difference was not statistically significant. The DLR models showed improved uncertainty estimates compared to the HCR models, suggesting their potential generalizability with unseen dataset. In addition, regarding sensitivity—defined as the capability of a model to correctly predict cases not achieving a complete response after CRT–DLR models showed superior performance. Identifying patients who were unlikely to achieve complete remission is particularly important in cancer prognosis prediction, as an initial treatment failure may lead to severe consequences for cancer patients.

Incorporating clinical factors tended to improve the prediction performance of the HCR model. When using only MRI data, the DLR model showed a marginally better performance than the HCR model (AUC; 0.721 for DLR vs. 0.597 for HCR). However, when clinical factors were integrated into the MRI data, there was no significant difference between the HCR and DLR models, although the DLR model showed a higher AUC (0.782 for DLR vs. 0.676 for HCR). The lack of statistical significance might be attributed to the small number of patients in the test dataset. Another plausible reason could be that the DLR model using only MRI data may not require additional clinical data to improve its performance, possibly due to the comprehensive information embedded within the image data. Several clinical factors, such as FIGO stage, tumor size, and parametrial invasion, have been reported to correlate with the CRT response of cervical cancer. In clinical decision-making, physicians do not rely on a single piece of information. To arrive at a conclusive decision, information from different categories, including medical imaging, laboratory tests, physical examinations, histopathologic, and genomic results, is combined. Therefore, integrating these heterogeneously originated data might be pivotal, even in the case of radiomics prediction. Similar findings have been reported in other radiomic series^5–7^, where integrating clinicopathologic or genomic data enhanced prognosis prediction in various cancers. For example, in a study by Lao et al., combining deep features with clinical factors improved survival prediction performance in patients with glioblastoma multiforme^8^. Similar results have been reported by Wang et al., who noted that the integration of laboratory factors (serum AFP and AST) exhibited better prediction in terms of survival in patients with hepatocellular carcinoma^5^. In another study using a lung cancer dataset, Aerts et al. revealed that combining radiomic features with stage information improved the prognosis prediction^9^. Our study is in line with these series, and suggests the benefit of combining clinical factors and imaging features rather than using imaging features exclusively*.* Summarizing previous publications and our results, imaging features and clinical data may have complementary roles in prognosis prediction in oncology.

However, the methodology for combining different types of data was quite diverse in each study. In a study by Lao et al., the authors constructed a nomogram using radiomics signatures and clinical factors^8^, whereas Wang et al. built a random forest model using radiomics signatures and laboratory factors as inputs simultaneously^5^, which is similar to the process followed in this study for building the HCR model. Other studies implemented a similar method that Wang et al. adapted, wherein image-generated radiomics features and clinical factors were fed into a regression model to predict the survival outcomes of patients with lung cancer^6,7^. In contrast to these studies, instead of dividing the feature extraction process and machine learning model building process, the DLR analysis performed herein leveraged optimized features via a data-driven approach. To build the DLR model, we combined the convolutional neural network (CNN) architecture for image feature extraction and the fully-connected layer for target task (i.e., classification). Subsequently, the entire system was trained using a learning algorithm, called backpropagation, in an end-to-end manner to minimize classification error. Backpropagation is an application of chain rule in calculus, particularly for training deep neural network; it calculates gradient of error with respect to entire weights of neural network. By backward propagating the gradients of error with the chain rule, all weights were updated with the gradient descent algorithm for finding a minimum of a function. Our DLR model extracted compact feature vectors that represent both imaging and clinical information simultaneously; subsequently, weights were updated to obtain optimized feature vectors to minimize errors. Moreover, because the feature extraction process was optimized by the learning algorithm in our DLR model, the effort on design choices for feature extraction were considerably reduced. In addition, as demonstrated in our experiments, the DLR model using MRI data tended to have superior performance compared to the HCR model using MRI data.

The choice of deep learning algorithm is a critical decision in radiomics research. We trained our CNN model using the transfer learning method^10^, which can consolidate general knowledge in large-scale data into specific new target tasks. Practically, the ImageNet pre-trained CNN can produce general feature representations from the natural images. Therefore, transfer learning with the pre-trained CNN has been widely applied to varied vision tasks, including object detection^11,12^, semantic segmentation^13,14^, and video recognition^15–17^. Medical image analysis is not an exception. Transfer learning with pre-trained CNNs is becoming popular in medical image classification tasks^18–23^. Huynh et al.^18^ used this approach for a breast tumor classification study. They pre-trained CNN with AlexNet’s architecture to extract deep learning features; subsequently, features from each layer were used to train the SVM classifier. Similarly, in a lung cancer dataset study by Paul et al., the authors pre-trained a CNN to extract deep features and built various machine learning models to predict survival. In our study, we employed one of the most popular CNN architecture, Residual Network (ResNet)^24^ proposed by Microsoft, as a backbone network. The ResNet won the 2015 ImageNet Large Scale Visual Recognition Challenge (ILSVRC) with a significantly improved error rate of 3.6%, essentially surpassing human performance. It enabled super-deep architecture by reducing the vanishing gradient phenomenon using skip connection (or residual connection) that allows gradient to always flow across extremely deep networks, improving performance significantly.

One of the challenges in adapting the DLR prediction model in the clinical scenario is its poor interpretability, which is the so-called “black box”. The interpretability of the DLR model refers to the recognition of which feature contributes to the decision making and how much. This helps both improve the model by knowing what exactly is going on in the neural network model and detect the failure points of the model. In our study, because the incorporation of clinical factors improved model performance in the DLR model, SHAP analysis was performed to reveal which clinical factor was more important for the CRT response prediction. Among the three clinical factors incorporated into the model, FIGO stage was the most important, followed by tumor size and HPV infection status. Recent studies have shown that HPV DNA negativity is associated with a poor prognosis^25–27^. Although HPV infection is an established etiology of cervical cancer, some patients unexpectedly show negative HPV test results, as in our study. HPV infection status was not a significant predictive factor for CRT response in the chi-square test or the most important factor in the SHAP analysis in our study. Our findings seem to contrast with those of studies reporting treatment outcomes of HPV-positive oropharyngeal cancer^28–30^. Patients with HPV-positive oropharyngeal cancer have shown superior recurrence-free survival and favorable prognosis compared with HPV-negative patients. Nonetheless, the SHAP analysis performed in this study was a tool for comparing the relative importance between factors. Thus, we are hesitant to make a definitive statement regarding the importance of HPV infection status. We report that the relative impact of FIGO stage was larger than that of primary tumor size and HPV infection status in our DLR model.

The limitations of our study include the potential selection bias associated with its retrospective nature and relatively small number of patients, which might lead to the lack of statistical difference. However, we attempted to minimize selection bias by including all consecutive cases that were homogenously treated according to a consistent protocol within an institution. Another limitation is that the data presented here were from a single institution, and external validation could not be performed. Therefore, generalizing the prediction model to an unseen dataset can be difficult. Furthermore, variability in scanners might influence the robustness of models^31,32^. Nevertheless, it is noteworthy that in real world scenario, it is common for different patients to acquire imaging examination by variable scanners, thus we still believe our findings can provide useful information for future studies. In addition, regarding HPV testing results, approximately 30% of the patients were missing, which may negatively impact the reliability of our prediction model.

In conclusion, both HCR and DLR models could predict CRT responses in patients with LACC. The integration of clinical factors into radiomics prediction models tended to improve performance in HCR analysis. However, further external validation using a larger, unseen dataset is required before clinical application in the future.

## Methods

### Study population

**Figure 4:**
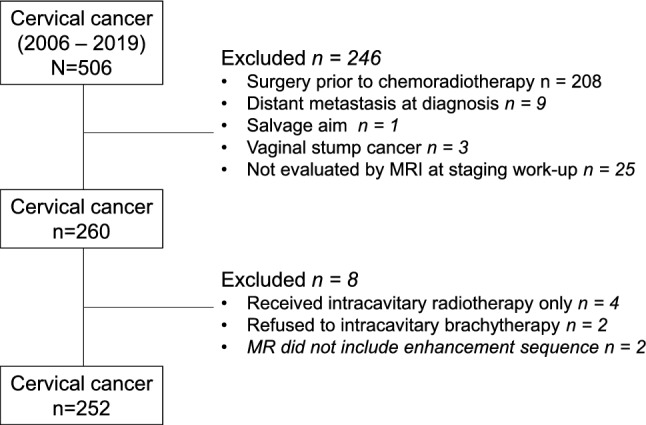
Flowchart of patient inclusion.

We retrospectively reviewed the medical records of 506 consecutive patients with cervical cancer treated with CRT at our institution between 2006 and 2019. The institutional review board of Kyungpook National University Chilgok hospital approved this study and waived the requirement for informed consent because anonymized data were used retrospectively (IRB No. KNUCH 2017-06-032). Among records initially screened, 246 patients were excluded for the following reasons: 208 underwent surgery before CRT, 9 had distant metastases at diagnosis, 3 were diagnosed with vaginal stump cancer, 1 received CRT for salvage purposes, and 25 were not evaluated by pretreatment MRI. Patients who underwent upfront surgery prior to radiation do not have any gross lesions suitable for imaging analysis. In addition, patients with distant metastases were treated with palliative aim of treatment to relieve cancer-related symptoms. Of the remaining 260 patients, 4 were treated with ICR alone, 2 refused ICR, and contrast-enhanced images were not taken for 2 patients. Finally, 252 patients were included in the analysis (Fig. 4). The dataset was randomly divided into two independent groups for the training (176 patients) and test (76 patients) datasets to get an equal frequency of cases (chemoradiotherapy response) in each dataset. The partitioning was done using ‘createDataPartition’ function from the ‘caret’ package in R. Contrast-enhanced T1-weighted fast spin-echo (FSE) images (CE-T1WI) and T2-weighted FSE images (T2WI) were obtained using various MR scanners. Baseline patient characteristics were collected from the electronic medical records. Before image segmentation, the patient-sensitive information was anonymized. Detailed information about image acquisition process was described in Supplementary material.

### Treatment characteristics and response evaluation

All patients were treated with EBRT and ICR with concurrent chemotherapy. EBRT was delivered to the entire pelvis using a three-dimensional (3D) conformal radiation therapy four-field box technique (1.8 Gy daily fractions, 5 times a week, for a total dose of 45 Gy). A parametrial boost of 10 Gy in 5 fractions was additionally administered to patients with parametrial involvement. ICR was delivered twice a week in five fractions with a fractional dose of 6 Gy. Weekly cisplatin at a dose of 40 mg/m^2^ was administered during radiotherapy. Patients were divided into complete response (CR) group or non-CR group according to the CRT response which was assessed 3 months after CRT by pelvic MRI and biopsy using the Response Evaluation Criteria in Solid Tumors (RECIST) version 1.1^33^. A total 195 out of 252 patients (77.4%) met the criteria of complete response.

### Radiomics modelling

**Figure 5:**
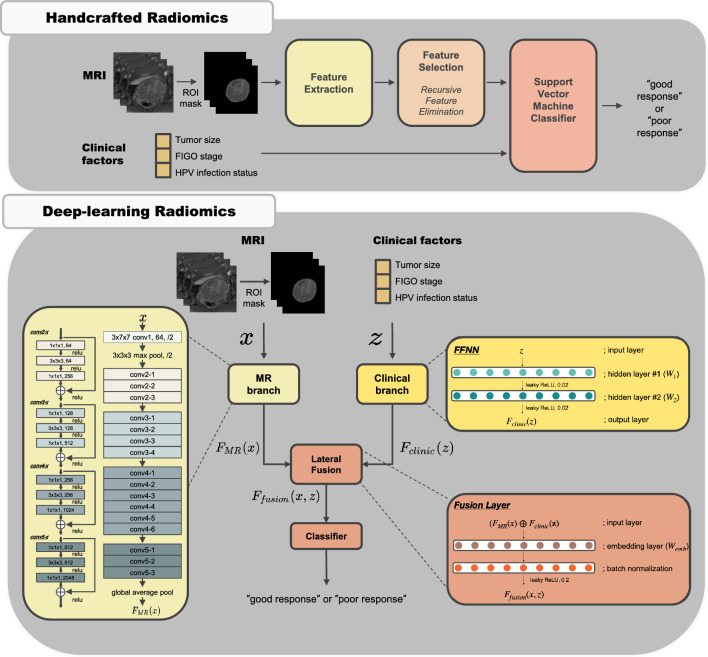
Study pipeline and model architecture of handcrafted radiomics (HCR) (top) and deep-learning radiomics (DLR) analysis (bottom). The proposed DLR model uses clinical factors as auxiliary inputs, along with MRI data. The MR branch extracts imaging features from three-dimensional (3D) MRI scans using an I3D network. The clinical branch converts clinical factors into a higher-dimensional vector using a feed-forward neural network (FFNN) with two layers. In the final step, these two representations are merged judiciously in the lateral fusion layer.

The key steps of the radiomics pipelines are illustrated in Fig. 5. The HCR pipeline included segmentation of tumor, feature extraction, feature selection, model building, and model validation. Primary tumor was semi-manually segmented on axial CE-T1WI and T2WI by two radiation oncologists (S.H and J.K) using the Eclipse treatment planning system, version 13.7 (Varian Medical Systems, Palo Alto, CA, USA). Clinical factors and HCR features were fed into SVM models. Among potential clinical factors, the three clinical factors (tumor size, FIGO stage, and HPV status) integrated into models to reduce dimensionality. Age was excluded due to its weak correlation with CRT response and the pathologic type was excluded owing to the highly imbalanced class proportion (Supplementary Table 2). Supplementary material provides a detailed description of how to carry out modeling process.

**Figure 6:**
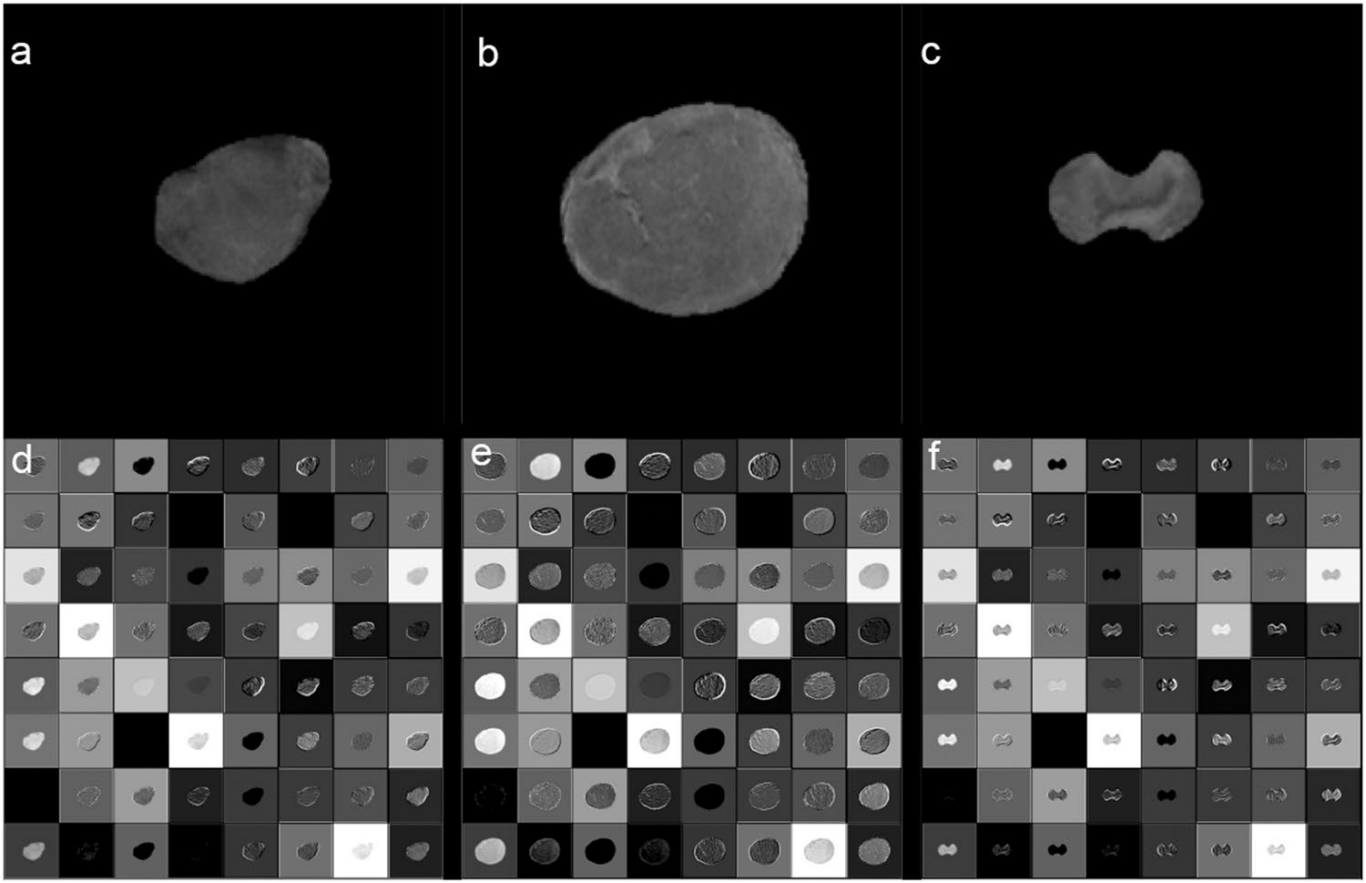
The visualization of features maps from the first convolutional layer of the cervical tumor at superior (**a**, **d**), middle (**b**, **e**), and inferior level (**c**, **f**) on T2-weighted MR images. Notice that the model exhibits responses to low-level visual elements such as edges and textures. These elements are crucial as they serve as foundational building blocks for recognizing complex patterns within images. Edges often represent boundaries between different objects or regions, potentially identifying the morphological characteristics of tumors in this case. Textures may provide information about tumor heterogeneity. By combining these basic elements, our model could capture more complex patterns associated with tumors.

The pipeline of DLR model consists of two branches: imaging and clinical factor branches. In the imaging branch, an inflated 3D (I3D) CNN was adopted as the base model. The I3D CNN can capture spatio-temporal information in 3D images. It extends capabilities of 2D CNNs into three dimensions (width, height, and depth), allowing it to consider the volumetric context and thereby better understand the depth dimension. This is particularly critical in medical imaging analysis where structures of interest may span across several slices or frames. The backbone network of our I3D CNN model was an ImageNet pre-trained two-dimensional CNN (ResNet-50) (Supplementary Table 3). Each pre-trained two-dimensional (2D) convolutional kernel with a spatial dimension of *k* × *k* was inflated, which implies that it was repeatedly stacked *l* times to process each 3D voxel of *l* × *k* × *k* (Fig. 6)*.* By applying inflated kernels on 3D images, the knowledge learned from the large-scale 2D image dataset (e.g., ImageNet) was transferred into our medical 3D image dataset, which is known as the transfer learning approach^10^. However, in transfer learning, when the target dataset is small and the number of parameters is large, fine-tuning the entire network may result in overfitting. Therefore, in this study, the I3D networks were fixed. Essentially, I3D was used for visual feature extraction, and its output did not change during the training. Herein, conv5_x feature maps that corresponded to the outputs of the last convolution blocks were extracted and average pooling was applied to obtain the imaging feature vector ${F}_{img}$.

In the clinical branch, a feed-forward neural network (FFNN), which consists of clinical factors, including tumor size, FIGO stage, and HPV infection status, was used. The following steps demonstrate its working. (i) Extract a bag of clinical factors, (ii) select the three most important features with the LASSO algorithm, and (iii) forward selected features to the FFNN. The clinical factor branch was inserted as an auxiliary input in the imaging branch to combine the imaging and clinical factor data. Consequently, a fusion layer, defined as I3D-fusion, was generated. A SHAP analysis was performed to identify the contribution of each clinical factor in the DLR model^34^. The SHAP value for each feature represents the average marginal contribution of a feature across all possible combinations of features. In other words, it quantifies how much each factor changes our prediction on average when it is included. The mean absolute SHAP values for each clinical factor were evaluated using the test dataset. The detailed methodology of HCR and DLR models is described in Supplementary material.

For the SVM classifier, hyperparameters were optimized through Scikit-learn, a Python machine learning library. A fivefold cross-validated grid search obtained ‘C’: 1000; ‘gamma’: 0.001; ‘kernel’: ‘rbf’ as the best parameters. For the CNN model, we used Optuna (https://optuna.org/), a Python library for hyperparameter optimization. A total of 100 trials were conducted with random hyperparameter settings, and the configuration that yielded the lowest validation loss was selected. Following hyperparameters were selected as the best hyperparameters: learning rate: 10^–3^; the number of hidden units: 25; batch size: 8. The hyperparameter search spaces are detailed in Supplementary Table 4. Python (v3.6.8) was the main programming language used. The proposed model was implemented with DLR framework PyTorch (v1.7.1). For ImageNet pre-trained 2D ResNet, ResNet50 implemented in TorchVision v.0.8.2 was used. A single NVIDIA TITAN RTX GPU (24 GB) was used for DLR analysis. We assessed the model performance using an open-source performance test tool for PyTorch model (https://github.com/sovrasov/flops-counter.pytorch). This tool measures the model’s Multiply-Accumulate (MAC) operations, which are the number of floating-point multiplication and addition operations in neural networks, as well as the number of parameters. For the DLR (MR model), the computational complexity was 160.3 GMac with 525,570 parameters and the inference speed was 48.747 ms (SD, 1.951) per run for DLR (MR) model. For DLR (MR + CF) model, the computational complexity was 160.3 GMac with 546,370 parameters and the inference speed was 49.593 ms (SD, 1.910) per run. Our code is made available open-source along with our experimental results at https://github.com/youhs4554/radiomics_CRT.

### Uncertainty of model predictions

In machine learning, uncertainty refers to the degree of confidence with which a model makes predictions. This aspect is particularly crucial in classification problems where an incorrect prediction can have significant consequences, such as in medical applications like ours. Quantifying the uncertainty allows us to assess the reliability of these predictions, thus highlighting their importance. We evaluated uncertainty using the Brier score^35^, which has been used in many studies^36^ to quantify uncertainty. The Brier score is an evaluation metric used to measure the accuracy of predicted probabilities in binary classification problems. The low brier score (i.e., close to zero) indicates that the model has high confidence in the predicted probabilities and they are in good agreement with the actual distributions, so we can conclude that the uncertainty is low and the model's prediction is trustworthy.

### Statistical analysis

All statistical tests were two-sided, and a *p*-value of < 0.05 was considered significant. Model performance was measured by performing receiver operating characteristic (ROC) analysis and calculating the area under the curve (AUC)^37,38^. ROC curve plots the true-positive rate and false-positive rate corresponding to all possible binary classification that can be formed from the continuous biomarker. The AUC is a measure of the accuracy of the test. A perfect test will have a value of 1.0, while a value of 0.5 suggests the prediction results is no better than random guess. Additionally, balanced accuracy, sensitivity, specificity, positive predictive value, negative predictive value, and F1-score were measured. The predictive performance of HCR and DLR models were compared using net reclassification improvement (NRI) and integrated discrimination improvement (IDI) indices^39,40^. The optimum cutoff of the DLR model was determined by maximizing the Youden index in the training dataset. To compare potential factors affecting CRT response, Student’s t-tests and Pearson’s chi-square tests were used to analyze continuous and categorical variables, respectively^41^. Statistical analyses were performed using R version 3.6.3 (R Foundation for Statistical Computing, Vienna, Austria). The R packages “caret”, “glmnet”, “pROC”, and “predictABEL” were used for analysis.

### Supplementary Information

Supplementary Information 1.

Supplementary Tables.

## Data Availability

The datasets generated and/or analyzed during the current study are not publicly available due to the privacy protection policy of personal medical information at our institution, but are available from the corresponding author on reasonable request.
