# Supplementary material for: Comparing deep learning and handcrafted radiomics to predict chemoradiotherapy response for locally advanced cervical cancer using pretreatment MRI
Source: Sci Rep. 2024 Jan 12;14:1180. doi: 10.1038/s41598-024-51742-z (PMC10786874; doi:10.1038/s41598-024-51742-z)
Supplement: Supplementary file 1 — Supplementary Information 1. [file 41598_2024_51742_MOESM1_ESM.pdf]

Supplementary Material

Comparing deep learning and handcrafted radiomics to predict chemoradiotherapy response for locally advanced cervical cancer using pretreatment MRI

Image acquisition

Contrast-enhanced T1-weighted fast spin-echo (FSE) images (CE-T1WI) and T2-weighted FSE images (T2WI) were obtained using various MR scanners. (Table A). In the CE-T1WI images, the median (range) repetition time (TR)/echo time (TE), slice thickness, field of view, and matrix size were 718 ms (2.9–3460)/11 ms (1.24–99.0), 5 mm (3.0–10.0), 260 (190–380) × 300 (190–380) mm, and 512 × 512, respectively. In the T2WI images, the median TR/TE, slice thickness, field of view, and matrix size were 3470 (4.4–14331.7)/99 (70.0—136.8), 5 mm (2.5–10), 260 (190–352.6) × 260 (190–352.6) mm, and 512 × 512, respectively.

Radiomics model building

Handcrfated radiomics

Primary tumor was semi-manually segmented on axial CE-T1WI and T2WI by two radiation oncologists (S.H and J.K) using the Eclipse treatment planning system, version 13.7 (Varian Medical Systems, Palo Alto, CA, USA). For each patient, 400 imaging features were extracted from CE-T1WI and T2WI using a Laplacian of Gaussian (LoG) filter and wavelet filter using Pyradiomics 3.0 (1): (i) first-order features, (ii) gray-level co-occurrence matrix (GLCM) features, (iv) gray-level size zone matrix (GLSZM), (v) gray-level run-length matrix (GLRLM), (vi) neighboring gray-tone difference matrix (NGTDM), and (vii) gray-level dependence matrix (GLDM) features. The mathematical definitions and feature descriptions are available at https://pyradiomics.readthedocs.io/en/latest/. A

fixed bin width of 20 was used. For image processing and feature calculation, the guidelines stated by the Image Biomarkers Standardization Initiative were followed (2). To normalize the image intensity from different MR units, intensity normalization was performed as a relative value (between the minimum and maximum values in the ROI) (3). The MR scans of the patients were resampled to slice thicknesses of 5 mm and pixel space of 0.4 mm × 0.4 mm.

Feature selection comprised the following two steps. First, logistic regression was performed for each radiomic feature to filter the features. Features with $p < 0.05$ underwent an additional selection step. Second, recursive feature elimination (RFE) was used to select the final features to build a prediction model, with five-fold cross-validation. A support vector machine (SVM) classifier with a five-fold cross-validation was used to generate a prediction model in the training dataset. During the classifier training, the hyperparameters were optimized using a grid search. The trained SVM model was validated using a test dataset. Three clinical factors, including tumor size, FIGO stage, and HPV infection status, were used to build the models as well as MRI radiomic features. Feature selection and ML modeling were implemented using the scikit-learn library in Python (3.7.6).

The architecture of CNN model

Preprocessing

For the DLR modelling, image frames were extracted from MRI scans as a preprocessing step. Typically, when training 3D convolutional neural network (CNNs), matching the number of frames across examples is required for efficient batch processing. To this end, shorter scans were padded with zero frames to match the max length of 77 scans. Pixel spacing was resampled to 0.4297 cm × 0.4297 cm × 2.5 cm. ROIs were used to train the models using the same ground-truth masks that had been used in HCR analysis. Each image was resized into 224 × 224 pixels, and divided by the mean pixel value of 127.5 to be normalized.

In our study, the CNN model consisted of two main components: imaging and clinicopathological branches. We created separate deep learning models and subsequently fused.

Imaging branch

The imaging branch is a convolutional model that can be applied to multiple 2D MR image scans. It can make decisions using only the imaging data. Let the feature map $\Phi(x)$ have dimensions of L × H × W × C, where L, H, W, and C are the axial length, height, width, and channels, respectively. Intuitively, the spatial dimension H × W captures spatial patterns, such as geometry and texture, which are related to the appearance of objects, whereas the axial dimension L captures the relations between the spatial planes. To obtain a global feature vector for a given volume, we simultaneously aggregated the spatial and axial information at the same time via global average pooling, which applied an L × H × W average filter to the feature map $\Phi(x)$.

Formally, the k_{th} element of the resulting feature vector F_{img} (x) for a given input x can be written as

$$F_{img}^{k} = \frac{1}{|\Phi^{k}|} \sum_{\phi \epsilon \Phi^{k}} \phi \, , for \;\; 1 \leq k \leq C,$$

where $\Phi^{k} \in R^{L \times H \times W}$ denotes the kth channel of the feature map $\Phi(x)$. Finally, the vector $F_{MR}(x)$ with dimension C was fed into a single fully connected classification layer.

Clinicopathologic branch

The clinicopathological branch was a simple feedforward neural network (FFNN). This branch consists of clinical factors, including tumor size, FIGO stage, and HPV infection status. The steps of its working mechanism are as follows. (i) Extract a bag of clinical factors, (ii) select the three most important features with the LASSO algorithm, and (iii) forward selected features to FFNN.

Formally, let F_c be the clinicopathological branch and z be a clinicopathological vector with m dimensions (in our case, m = 3). For a given z, we can obtain a clinical representation by (the bias term has been omitted for simplicity):

$$F_c(z) = h_2(z) = a(W_2^T h_1) = a(W_2^T \left(a(W_1^T z)\right)),$$

where $W_1 \in R^{m \times h_{cf}}$ is the weight matrix of 1st hidden layer (h_1) and $W_2 \in R^{h_{cf} \times h_{cf}}$ is the 2nd hidden layer (h_2). It produces an output vector $F_c(z) \in R^{h_c \times 1}$.

The $a(\cdot)$ refers to the Leaky ReLU (4) activation function, whose negative slope is set to 0.02. It is formulated as

$$a(s) = max\{0.02s, s\},$$

where s is the input to the activation function, that is $s = W^T x + b$. The number of output nodes was set to 1/32 of the channels of the imaging branch outputs (thus, $h_{cf} = C/32$) to prevent the imaging branch from being overwhelmed by the clinicopathologic branch.

Lateral fusion

The two constructed branches were laterally fused to generate an I3D-fusion network. In particular, $F_{img}(x)$, the global average pooling of the last output of ResNet-50 'conv5_x', was concatenated with the $F_c(z)$. The new model, $F_{fusion}(x; z)$, learned a compact (with $h_{emb} = C/8$ dimension) vector representation simultaneously containing both imaging and clinicopathologic data by judiciously reducing the concatenated vector dimension in embedding layer, which refers to a single fully-connected layer whose weight matrix was W_{emb}. The proposed lateral fusion layer is formulated as follows.

$$F_{fusion}(x; z) = emb\big(F_{MR}(x) \oplus F_{cf}(z); W_{emb}\big),$$

where $\oplus$ represents a fusion operation (e.g., concatenation). For $emb(\cdot)$, the batch normalization layer was located before the activation function, and the slope of the leaky ReLU for the negative input was set to 0.2 (steeper than 0.02). Finally, it was fed into a fully connected classification layer.

Loss function

The α-balanced form of the focal loss function, proposed by Lin et al. (5), was used to deal with the imbalance in class distribution. The formula is as follows.

$$\mathrm{FL}(p_t, y) = -\alpha_t (1 - p_t)^{\gamma},$$

$$\text{where } p_t = \begin{cases} p & if\ \ y = 1 \\ 1 - p & otherwise, \end{cases}$$

where $y \in \{0, 1\}$ refers to the binary ground-truth class, $p \in \{0, 1\}$ is the estimated probability for class with label $y = 1$, and $\alpha_t \in \{0, 1\}$ is the weighting factor defined as the inverse class frequency of each ground-truth class label, and γ is the tunable focusing parameter. In this study, $\gamma = 2$ was used. A modulating factor $(1 - p_t)^{\gamma}$ could down-weight well-classified examples with high p_t whereas the loss contribution could be increased with a small p_t. For positive examples with a small p_t owing to their scarcity during training, rare positive examples could further affect the model.

Implementation details

The backbone network of our I3D CNN model was an ImageNet pre-trained two-dimensional CNN (ResNet-50). The SAM optimizer (6) was used to prevent training loss from converging to sharp minima and to find smooth minima to improve the generalization performance. An initial learning rate was 0.001 and batch size was 8. CNN models were trained for 25 epochs with learning rate decay, which was reduced by 0.75 times at every 5 epochs. To reduce the variance in the final neural network model, the model-averaging ensemble method was adopted. Instead of training the same model on the same dataset repeatedly, each ensemble member was made to observe a different subset of the training dataset during training to reduce overfitting. First, the training dataset was split into five folds, and each fold was split into training and validation sets again. Subsequently, each ensemble member was fitted with the training dataset for each fold and the validation loss was measured. To further prevent overfitting, the early stop method, wherein the training process was stopped if the validation loss was not improved within 10 epochs, was used. These steps were repeated for all the folds to obtain an 'expert ensemble

member' for each fold. After completing the training of all expert members, multiple prediction scores by expert members on the holdout test dataset were averaged to evaluate the final results.

References

1. van Griethuysen JJM, Fedorov A, Parmar C, Hosny A, Aucoin N, Narayan V, Beets-Tan RGH, Fillion-Robin J-C, Pieper S, Aerts HJWL. Computational radiomics system to decode the radiographic phenotype. Cancer Research 2017;77(21):e104-e107. doi: 10.1158/0008-5472.can-17-0339

2. Zwanenburg A, Vallières M, Abdalah MA, Aerts H, Andrearczyk V, Apte A, Ashrafinia S, Bakas S, Beukinga RJ, Boellaard R, Bogowicz M, Boldrini L, Buvat I, Cook GJR, Davatzikos C, Depeursinge A, Desseroit MC, Dinapoli N, Dinh CV, Echegaray S, El Naqa I, Fedorov AY, Gatta R, Gillies RJ, Goh V, Götz M, Guckenberger M, Ha SM, Hatt M, Isensee F, Lambin P, Leger S, Leijenaar RTH, Lenkowicz J, Lippert F, Losnegård A, Maier-Hein KH, Morin O, Müller H, Napel S, Nioche C, Orlhac F, Pati S, Pfaehler EAG, Rahmim A, Rao AUK, Scherer J, Siddique MM, Sijtsema NM, Socarras Fernandez J, Spezi E, Steenbakkers R, Tanadini-Lang S, Thorwarth D, Troost EGC, Upadhaya T, Valentini V, van Dijk LV, van Griethuysen J, van Velden FHP, Whybra P, Richter C, Löck S. The image biomarker standardization initiative: Standardized quantitative radiomics for high-throughput image-based phenotyping. Radiology 2020;295(2):328-338. doi: 10.1148/radiol.2020191145

3. Shinohara RT, Sweeney EM, Goldsmith J, Shiee N, Mateen FJ, Calabresi PA, Jarso S, Pham DL, Reich DS, Crainiceanu CM, Australian Imaging Biomarkers Lifestyle Flagship Study of A, Alzheimer's Disease Neuroimaging I. Statistical normalization techniques for magnetic resonance imaging. Neuroimage Clin 2014;6:9-19. doi: 10.1016/j.nicl.2014.08.008

4. Xu B, Wang N, Chen T, Li M. Empirical evaluation of rectified activations in convolutional network. 2015. doi: 10.48550/ARXIV.1505.00853

5. Lin TY, Goyal P, Girshick R, He K, Dollár P. Focal loss for dense object detection. 2017 IEEE

International Conference on Computer Vision (ICCV)2017; p. 2999-3007.

6. Lundberg SM, Lee S-I. A unified approach to interpreting model predictions. Proceedings of the 31st International Conference on Neural Information Processing Systems: Curran Associates Inc., 2017; p. 4768–4777.

Table A. List of MR scanners used in the study

Manufacturer	Model	T	N (%)
Siemens	Magnetom Vision	1.5	96 (38.1)
Siemens	Magnetom Avanto	1.5	59 (23.4)
GE	Discovery MR750	3.0	40 (15.9)
GE	Signa Excite	3.0	16 (6.3)
Siemens	Symphony	3.0	14 (5.6)
Siemens	Skyra	3.0	12 (4.8)
Siemens	Sonata	1.5	3 (1.2)
Siemens	Spectra	3.0	3 (1.2)
Siemens	Verio	3.0	3 (1.2)
Philips	Achieva	3.0	3 (1.2)
Philips	Ingenia	3.0	2 (0.8)
Siemens	Biograph	3.0	1 (0.4)
