# Supplementary material for: Comparing deep learning and handcrafted radiomics to predict chemoradiotherapy response for locally advanced cervical cancer using pretreatment MRI
Source: Sci Rep. 2024 Jan 12;14:1180. doi: 10.1038/s41598-024-51742-z (PMC10786874; doi:10.1038/s41598-024-51742-z)
Supplement: Supplementary file 2 — Supplementary Tables. [file 41598_2024_51742_MOESM2_ESM.docx]

Supplementary Table 1. Selected handcrafted radiomic features by recursive feature elimination in training dataset.

| **MR sequence** | **Feature name** |
| --- | --- |
| T1 | LoG glcm_SumSquares  LoG glszm_SmallAreaHighGrayLevelEmphasis  LoG glszm_ZonePercentage  wavelet.LLH_gldm_DependenceNonUniformity  wavelet.HLL_glszm_HighGrayLevelZoneEmphasis  wavelet.HLL_glszm_LowGrayLevelZoneEmphasis  wavelet.LLL_gldm_SmallDependenceHighGrayLevelEmphasis  wavelet.LLL_glszm_ZonePercentage |
| T2 | Shape_Maximum3DDiameter  glrlm_RunLengthNonUniformity  glszm_ZoneVariance  LoG glcm_MaximumProbability  wavelet.LLH_glcm_ClusterShade  wavelet.HHL_gldm_HighGrayLevelEmphasis  wavelet.HHL_gldm_LowGrayLevelEmphasis  wavelet.HHL_glszm_SmallAreaEmphasis  wavelet.HHL_glszm_SmallAreaLowGrayLevelEmphasis  wavelet.LLL_firstorder_Skewness  wavelet.LLL_glszm_LargeAreaHighGrayLevelEmphasis  wavelet.LLL_glszm_ZoneVariance |
| LoG, Laplacian of Gaussian filter; glcm, gray level co-occurrence matrix; glszm, gray level size zone matrix; gldm, gray level dependence matrix; glrlm, gray level run length matrix. | |

Supplementary Table 2. Potential factors related to chemoradiotherapy response.

| Characteristic | N | Complete remission | Not complete remission | p-value |
| --- | --- | --- | --- | --- |
| Age  ≤57  >58 | 131  121 | 102 (77.9 %)  93 (76.9 %) | 29 (22.1 %)  28 (23.1 %) | 0.969 |
| Pathology  Squamous cell carcinoma (SCC)  Non-SCC | 231  21 | 182 (78.8 %)  13 (61.9 %) | 49 (21.2 %)  8 (38.1 %) | 0.100 |
| Tumor size (mm)  <50  ≥50 | 134  118 | 103 (76.9 %)  92 (78.0 %) | 31 (23.1 %)  26 (22.0 %) | 0.954 |
| FIGO Stage  IIB-IIIB  IIIC1-IVA | 198  54 | 151 (76.3 %)  44 (81.5 %) | 47 (23.7 %)  10 (18.5 %) | 0.529 |
| HPV infection status  Positive  Negative | 133  39 | 106 (79.7 %)  35 (89.7 %) | 27 (20.3 %)  4 (10.3 %) | 0.231 |

Supplementary Table 3. Convolutional network architecture for MR branch implementation considered in our experiments. This architecture is an inflated version of ResNet-50. The dimensions of 3D output size and kernels are in $L\times H\times W$, followed by the number of channels. Residual blocks shown in brackets are repeated as a number on each side. And in last fully-connected (fc) layer classifies with 2048-d vector. In our experiment, the input was $77\times224\times224$.

| Layer | | | Output size | |
| --- | --- | --- | --- | --- |
| conv1 | $3\times7\times7$, 64, stride 2 | $L\times112\times112$ | |  |
| conv2_x | $3\times3\times3$ max pool, stride 2 | $L/2\times56\times56$ | |  |
|  | $\left[ \begin{aligned} 1\times1\times1, 64 \\ 3\times3\times3, 64 \\ 1\times1\times1, 256 \end{aligned} \right]\times3$ |  |  |  |
| conv3_x | $\left[ \begin{aligned} 1\times1\times1, 128 \\ 3\times3\times3, 128 \\ 1\times1\times1, 512 \end{aligned} \right]\times4$ | $L/4\times28\times28$ | |  |
| conv4_x | $\left[ \begin{aligned} 1\times1\times1, 256 \\ 3\times3\times3, 256 \\ 1\times1\times1, 1024 \end{aligned} \right]\times6$ | $L/8\times14\times14$ | |  |
| conv5_x | $\left[ \begin{aligned} 1\times1\times1, 512 \\ 3\times3\times3, 512 \\ 1\times1\times1, 2048 \end{aligned} \right]\times3$ | $L/16\times7\times7$ | |  |
| Global average pool, 204-d fc, softmax | | | # classes | |

Supplementary Table 4. Hyperparameter search space for model training.

| Algorithm | Parameters | Search Space |
| --- | --- | --- |
| Support vector machine classifier | kernel | linear, rbf, sigmoid |
|  | *C* | [1–1000] |
|  | gamma | [0.0001–0.01] |
| Convolutional neural network | learning rate | [0.0001–0.01] |
|  | number of hidden units of clinical branch | [32, 512] |
|  | batch size | [4–32] |
